# 

**Published:** 1891-04

**Authors:** 


### CONTENTS—APRIL, 1891.—VOL. VI., NO. 10.

ORIGINAL ARTICLES—
Observations on the Omœba Coli in Dysentery and Abscess of the Liver. Geo. Dock, M. D ................ 419
Management of the Pedicle in Ovarian Cystomata. A. C. Walker, M. D .................................. 432
Improper Use of Antipyrin, etc. S. L. Keown, M. D. ..434
Hæmatinuria vs. Malarial Hæmaturia. F. S. Young, M.D. 438
Cancrum Oris. W. P. Fleming, M. D ................. 440
Tracheo-Laryngotomy. D. B. McGehee, M. D .......... 441
Medical Legislation—Quarantine Bill ................... 443
SOCIETY NOTES—
Meeting of Medical Societies ......................... 448
EDITORIAL—
Medical Legislation—the Quarantine Bill ............... 449
Pay-Day .............................................. 451
Dr. Dock's Article ................................... 451
The Pedicle .......................................... 452
Improper Use of Antipyretics ......................... 452
MEDICAL NEWS AND MISCELLANY—
Numerous items of news of interest to physicians ...... 452
PUBLISHERS' NOTES—
Having reference to advertisements, etc ............... 456

